# Does a transition in education equate to a transition in practice? Thai stakeholder’s perceptions of the introduction of the Doctor of Pharmacy programme

**DOI:** 10.1186/s12909-015-0473-4

**Published:** 2015-11-19

**Authors:** Teeraporn Chanakit, Bee Yean Low, Payom Wongpoowarak, Summana Moolasarn, Claire Anderson

**Affiliations:** School of Pharmacy, University of Nottingham, Nottingham, UK; School of Pharmacy, Faculty of Science, University of Nottingham Malaysia Campus, Semenyih, Selangor Darul Ehsan, Malaysia; Faculty of Pharmaceutical Sciences, Prince of Songkla University, Hat Yai, Songkhla Thailand; Faculty of Pharmaceutical Sciences, Ubon Ratchathani University, Warin Chamrap, Ubon Ratchathani Thailand

**Keywords:** Stakeholders, Perceptions, Transition, PharmD programme, Preceptors, Workforce, Thailand, A qualitative study

## Abstract

**Background:**

Pharmacy education and pharmacy practice are facing remarkable changes following new scientific discoveries, evolving patient needs and the requirements of advanced pharmacy competency for practices. Many countries are introducing or undertaking major transformations in pharmacy education. The Thai pharmacy curriculum has been changed from a 5-year BPharm and a 6-year PharmD to only a 6-year PharmD programme. Curriculum change processes usually involve stakeholders, including both internal and external educational institutions, at all levels. This study aims to understand the experiences and perceptions of stakeholders regarding the transition to an all-PharmD programme in Thailand.

**Methods:**

Semi-structured interviews were conducted in Thailand with 130 stakeholders (e.g., policy makers, pharmacy experts, educators, health care providers, patients, students and parents) from August-October 2013. The interviews were audio recorded, transcribed verbatim and analysed using an inductive thematic analysis.

**Results:**

Three main themes were derived from the findings: 1. influences on curriculum change (e.g., the needs of pharmacists to provide better patient care, the US-Thai consortium for the development of pharmacy education); 2. perceived benefits (e.g., improve pharmacy competencies from generalists to specialists, ready to work after graduation, providing a high quality of patient care); and 3. concerns (e.g., the higher costs of study for a longer period of time, the mismatch between the pharmacy graduates’ competency and the job market’s needs, insufficient preceptors and training sites, lack of practical experience of the faculty members and issues related to the separate licenses that are necessary due to the difference in the graduates’ specialties).

**Conclusions:**

This is the first study to highlight the issues surrounding the transition to the 6-year PharmD programme in Thailand, which was initiated due to the need for higher levels of competency among the nation’s pharmacists. The transition was influenced by many factors. Many participants perceived benefits from the new pharmacy curriculum. However, some participants were concerned about this transition. Although most of the respondents accepted the need to go forward to the 6-year PharmD programme, designing an effective curriculum, providing a sufficient number of qualified PharmD preceptors, determining certain competencies of pharmacists in different practices and monitoring the quality of pharmacy education still need to be addressed during this transitional stage of pharmacy education in Thailand.

## Background

Pharmacy education and pharmacy practice are facing remarkable changes following new scientific discoveries, evolving patient needs and the requirements for advanced pharmacy competency for current and future practices [1, 2]. Therefore, many countries are introducing or undertaking major transformations in pharmacy education [1, 2]. The World Health Organization (WHO), the United Nations Educational, Scientific and Cultural Organisation (UNESCO) and the International Pharmaceutical Federation Education Development Team (FIP*Ed*) all aim to improve global pharmacy education and have developed a “needs-based education model” [1, 3–6]. The model stipulates that pharmacy education programmes must be designed to ensure that required competencies are achieved by all pharmacy graduates to deliver pharmacy services that meet the needs of national populations. Many countries have been upgrading their pharmacy degree programmes to the level of Doctor of Pharmacy (PharmD) [7–13]. However, literature exploring the adoption of such programmes in Asian countries is limited [14–16].

**Fig. 1:**
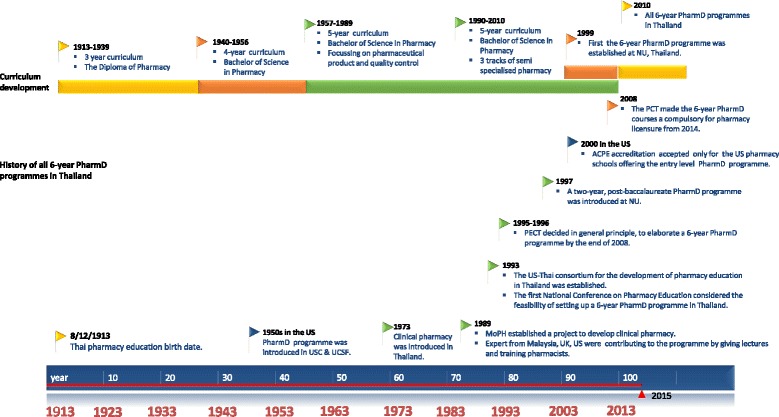
History of all 6-year PharmD programmes in Thailand

The initiation of modern pharmacy education in Thailand began in 1913 with a 3-year programme which extended to a 4-year programme in 1939 and then to a 5-year Bachelor in Pharmacy (BPharm) programme in 1957 [17–19]. In 1993, the US-Thai consortium for the development of pharmacy education in Thailand was established [19]. Thai pharmacy educators and pharmacy practitioners participated in this collaboration to develop the academic workforce that was needed. In 1999, the first of the Thai Doctor of Pharmacy (PharmD) programmes, which focused on patient care at the Faculty of Pharmaceutical Sciences, Naresuan University, was established [18, 20–22]. The details of the history of all 6-year PharmD programmes in Thailand are shown in Fig. 1 [17, 23–36].

From 1990–2010, the majority of Faculties of Pharmacy in Thailand offered a 5-year BPharm programme. The 5-year programme was divided into three tracks: pharmaceutical sciences, pharmaceutical care and social and administrative pharmacy; this allowed students to achieve more professional specialisation [18, 23, 37]. However, the new pharmacy graduates and employers felt that the graduates were still inadequately trained and were not prepared for post-graduation practice [28, 38].

Similar to other countries that were changing to an Entry-Level PharmD (ELPD) programme [39–46], unified support for the transition to an all- PharmD programme was also limited in Thailand [39, 45]. Pramyothin et al. [30] found that while most experts from hospitals and consumer protection sectors agreed with a 6-year programme with special tracks, advanced practitioners from the community pharmacy and industry areas preferred a 5-year programme with special tracks. Wongpoowarak et al. [47] found that most pharmacy graduates and employers felt that a 6-year PharmD was needed for both tracks (pharmaceutical care and pharmaceutical sciences); they also reported that while academic staff had mixed views regarding the appropriate duration of a pharmacy programme in pharmaceutical sciences, the majority (63 %) sided with 5 years as opposed 15 % and 22 % who preferred 4 years and 6 years, respectively. Another study which carried out a survey of pharmacists regarding curriculum changes found that most of the respondents did not agree that a 6-year programme would either decrease the pharmacy curriculum workload or improve the status of pharmacists to reach that of medicine and dentistry graduates (both also 6-year programmes). Most respondents agreed that the pharmacy curriculum should be producing generalists but that specialties should be studied in higher education. The majority of the respondents believed that the pharmacy curriculum should include both 5- and 6-year options, while about 20 % of respondents believed that only one the 6-year programme should be offered [48, 49].

Thai policy makers believed that a complete transition to the 6-year PharmD would meet the needs of the stakeholders by changing pharmacy competencies from generalists to specialists, resolve the issue of curriculum overload for the high-credit 5-year BPharm programme, and produce equal educational standards and outcomes and for the pharmacy profession on a national level [28, 38]. The Pharmacy Council of Thailand (PCT) announced that the 6-year PharmD programme would be compulsory for pharmacy licensure starting from year 2014 onwards; the BPharm programme was not offered after 2010 [18, 28, 30, 37]. All new pharmacists have to graduate from pharmacy schools accredited by the Pharmacy Council, with a 6-year PharmD degree based on the new 6-year curriculum, a move designed to provide a more comprehensive curriculum and in-depth knowledge and skills in each specific pharmacy area [17, 18, 37, 50]. The US PharmD programme, which focuses on patient care, has been adjusted to meet the context of the Thai health system. The new PharmD curriculum is divided into two tracks, a Pharmaceutical Care-PharmD (PC-PD) programme and an Industrial Pharmacy (formerly Pharmaceutical Sciences)-PharmD (IP-PD) programme [23, 37, 51–53]. It is still under discussion as to whether another track, Social and Administrative Pharmacy (SAP), should either be independent or included in the other two tracks [18, 23]. Pharmacy students in some faculties will select their specialty upon admission; in other faculties, students select their speciality programmes in the fourth year of the PharmD [37].

The transition has been implemented by a collaboration among regulatory bodies (e.g., PCT and government) and educational institutions [28]. However, debates and questions have been put forth on social networks about the need for a change to an all-PharmD programme in Thailand [48, 49]; among the questions asked were: ‘How would an all-PharmD programme produce graduates to meet the needs of various areas of pharmacy practices in Thailand’? ‘How to incorporate the diverse competencies of two specialised areas (PC-PD and IP-PD) into one professional license?’ [18, 22, 30, 48]. Additionally, the limited resources and capacities of academic and training institutions in developing countries that are changing to a PharmD programme have been discussed [5, 54].

Stakeholders are an important aspect of the “needs-based model” comprised by FIPEd’s Global Quality Assurance Framework because it is the stakeholders who dictate local or national needs [2, 55]. For example, regulatory bodies, such as government and the Pharmacy Council, are responsible for pharmacy practice. They have the duty to protect the wellbeing of the public by assuring that the pharmacy workforce receives appropriate education and training to deliver a standard quality of services [2]. Curriculum change involves and affects many stakeholders, including both internal and external educational institutions (e.g., students, faculty members, staff members, administrators, employers, employees, patients and the public) at different levels, different roles and also different expectations [2, 55–58]. As such, exploring the stakeholders’ perspectives and experiences regarding this transition is crucial. There has been very little evidence, to date, of such attempts in Thailand. Qualitative research can provide meaning and understanding and is ideally suited to explore a little researched area [59, 60]. Therefore, this paper reports on a qualitative study which was aimed to understand the experiences and perceptions of stakeholders regarding the transition to an all-PharmD programme in Thailand.

## Methods

This study was granted ethical approval from the Faculty of Sciences, University of Nottingham and the ethics authorities in Thailand (the Research Ethics Committee of Ubon Ratchathani University and the Research Ethics Committee of Buddhachinaraj Hospital). Semi-structured interviews were conducted with 130 stakeholders in the Thai language during 1 Aug - 20 Oct, 2013.

Triangulated data were collected from different type of stakeholder categories via interviews across four different regions (e.g., Central, North, North-East, and South) to check and establish validity [61–63] and also include sub-groups of each stakeholder category to further maximise sample variation [62, 64] (e.g. both administrators and non-administrators from educational institutions were targeted). Stakeholders groups included policy makers, pharmacy experts or representatives of the pharmacy profession’s associations (e.g., public and private hospital pharmacy, community pharmacy, industrial pharmacy, public health and consumer protection, marketing pharmacy), educators (e.g., public and private university), practicing pharmacists from different settings (e.g., tertiary hospital, community hospital, community pharmacy, private hospital, industrial pharmacy, consumer protection, pharmacy marketing, research and development), health care providers (doctors, nurses, pharmacy technicians), patients, students and their parents as well as members of the general population. Participants included those involved in the quality assurance of pharmacy education according to the FIP definition [2]. Consensus among the main researchers (TC, CA, PW) was reached with regards to the selection process and sampling of participants. Table 1 lists the inclusion criteria.

**Table 1:** The inclusion criteria and the selection of participants [2, 65–75]

Stakeholder group	Central	North	North-East	South	Total	Roles and importance in pharmacy education	Selection and recruitment	Method of involvement
1. Policy makers/regulatory bodies (n=10)								
-The Pharmacy Council of Thailand (PCT) is the regulatory body for pharmacists in Thailand.	3	0	1	1	5	-The PCT has a role to protect and maintain the wellbeing of the public by maintaining standards and public trust in pharmacy (e.g., setting standards for conduct, ethics, and competency, accrediting pharmacy degree programmes and pharmacy educational institutions, processing licensure examination and registration). They must assure that pharmacists receive appropriate education and training and are competent to deliver services.	-Policy makers, who had or still have roles in curriculum change ever since the issue of an all-PharmD was raised in 1993, were invited by email or letter and telephone at least one month prior to the interview date. -Eleven policy makers were invited to participate; one policy maker from the Pharmacy Council did not respond within the timeframe of the study.	Interview
-The Pharmacy Education Consortium of Thailand (PECT) has seventeen pharmacy educational institution members across Thailand.	1	1	3	0	5	-PECT aims to promote the advancement of pharmacy education, support the pharmacy professional practice, protect common interests among members, facilitate pharmacy student activities, and collaborative work with other professional organisations (e.g., PCT and Thai pharmacy associations). -PECT prepared a government scholarship to support faculty development and by 1993 PECT signed the first bilateral collaboration between US and Thai pharmacy schools, the US-Thai Consortium for the Development of Pharmacy Education in Thailand. This consortium opened opportunities for Thai academic members and students to be trained and study in the US.		
2. Pharmacy experts (n=13) -Pharmacy experts or representatives of professional pharmacy associations in Thailand -Hospital pharmacy (from both public and private hospitals) -Community pharmacy -Industrial pharmacy -Public health and consumer protection -Marketing pharmacy	1 2 3 3 1	0 0 0 0 0	1 1 0 0 0	1 0 0 0 0	3 3 3 3 1	Pharmacy experts and pharmacy practitioners are able to determine the competencies required to deliver the services that meet the needs of employers and customers.	-Pharmacy experts or representatives of professional associations were identified primarily through the public websites of their organisations, selected by researchers, and invited by email and telephone at least one month prior to the interview date. -Experts or representatives from pharmacy associations from five areas in Thailand (e.g., the Association of Hospital Pharmacy (Thailand), the Community Pharmacy Association (Thailand), the Thai Industrial Pharmacist Association, the Marketing Pharmacy Association Thailand) and the Thai Food and Drug Administration (FDA) as well as law and consumer protection area and experts from the Pharmaceutical Association of Thailand, who were current practitioners, well known and were accepted by pharmacists in their professional practice areas, were invited. -Sixteen experts or representatives of pharmacy organisations were invited to participate in this study; three experts (1 from a hospital, 1 from industry and 1 from marketing) did not respond within the timeframe of the study. n=13)	Interview
3. Academic staff (n=25) -Dean -Deputy dean (academic/professional development)	U^a^1 U2 2 1	U3 1 1	U4 1 1	U5 1 1	5 4	-Faculties of pharmacy and academic members have responsibilities in terms of the quality of education and value of investment in higher education for students and families. Faculty members committed to lifelong learning in their specialty and continuous improvement of their teaching skills, devote sufficient time to teaching along with research, administrative or academic services (e.g., clinical roles) and also have a duty to develop learning opportunities and encourage pharmacy students to participate. Faculty staff have influence on promoting learning environments, demonstrate professional responsibility, encourage and assist their students to assume responsibility for their learning and express concern regarding student development.	-The criteria to select the universities were based on the region of the university, type of ownership, type of programme, year established and feasibility of study. -Five universities (four public and one private university) were selected and invited and a letter requesting permission to conduct research with each faculty was sent to the dean. All faculty granted permission to conduct this research. -Deans, deputy deans of academic affair or deans in pharmacy professional development and faculty members were invited. -The details of the five faculties are as follows: •U1 (public) used to offer BPharm and then changed to PC-PD & IP-PD •U2 (private) used to offer BPharm and then changed to PC-PD & IP-PD •U3 (public) offering PC-PD •U4 (public) used to offer BPharm and then changed to PC-PD •U5 (public) used to offer BPharm and PC-PD and then changed to PC-PD & IP-PD	-Interview -Skype^b^ -Telephone interview^b^
Academic staff -Pharmaceutical care area -Industrial pharmacy/pharmaceutical sciences area -Social and administrative pharmacy area	1 2 1	2 1 1	2 1 2	1 1 1	6 5 5			
4. Students (n=9)	3	1	3	2	9	Students and their families invest finances and time to obtain a pharmacy degree and aim to learn within a pharmacy curriculum designed to prepare them for the profession. They have the right to expect that the PharmD programme has been sufficiently evaluated and meets the standards outlined by the PCT which should able to prepare them for a pharmacy professional career. Students should expect that the pharmacy curriculum is timely, practical and provide essential knowledge and skills for successful professional practice.	-Inclusion criteria were students who were currently studying in the final year of a 6-year PharmD programme because they had experience with the new 6-year PharmD programme or the students who were studying in the 5^th^ year if their faculties did not have 6^th^ year students during this study period (Aug-Oct 2013). Participation was voluntary. -Students were invited to participate by a researcher at the cafeteria or library of their faculty at least 1 day prior to an interview date.	-Interview -Telephone interview
5. Parents (n=4)	2	2	0	0	4	Parents play an important role in students’ university life (e.g., parental support in terms of facilitating personal, social, and academic development and finances). They are concerned about the accreditation of institutions for the mature development of their children and preparing their children for the profession.	-Inclusion criterion was a parent of the student who was currently studying in the final year of a 6-year PharmD programme. -Researcher asked the students about the possibility of contact their parents to participate in this study. -Most parents lived in other provinces. Therefore, parents were invited and interviewed by the researcher via telephone.	-Telephone interview
6. Pharmacists (n=30) -Tertiary hospital (Three pharmacists are the administrators) -Community hospital -Community pharmacy -Private hospital (One pharmacists is an administrator) -Industrial -Public health and consumer protection -Marketing -Research and development	2 0 2 1 1 0 2 0	3 0 0 0 0 0 0 0	4 4 2 1 0 2 0 2	2 0 1 0 1 0 0 0	11 4 5 2 2 2 2 2	Pharmacy experts and pharmacy practitioners are able to determine the competencies required to deliver services that meet the needs of the final user (e.g., patient, other health care providers and public).	Health care providers (doctors, nurses, pharmacists and pharmacy technicians), students and their parents were contacted directly at their workplace at least 1 day prior to an interview date. However, the patients (at two hospitals) and general population (at local supermarkets in urban and rural areas) were asked for their participation on the actual interview date, due to employing convenience sampling.	-Interview
7. Health care providers (n=18) -Physicians (Three physicians are the administrators) -Nurses (Two nurses are the administrators) -Pharmacy technicians	1 2 2	1 2 0	5 2 2	0 0 1	7 6 5	The pharmacy profession has embraced a patient-focused role and works closely with other health care professionals. Health care providers’ opinions are very important for the development of the pharmacy curriculum as multidisciplinary education, especially in terms of advanced practice experience or clinical clerkships, during which pharmacy students are exposed to other health care providers and learn how to become a member of a team after they graduate. In addition, health care provider opinions will explain how other health profession view the role of pharmacists in health care teams.	Health care providers (doctors, nurses, pharmacists and pharmacy technicians) were invited directly at their workplace at least 1 day prior to an interview date.	-Interview
8. Patients (n=14) from two hospitals (tertiary care hospital and community hospital) -In-Patient Department, IPD (n=5) -Out-Patient Department, OPD (n=9)	0 0	4 6	1 3	0 0	5 9	-Pharmacists work with patients to ensure that they receive the best outcomes from their medications. -They are the final users of pharmacy practitioners. Patients’ opinions are important to improve the quality of pharmacy services and enhance communication and expectations between patients and pharmacists, increase the quality of pharmacy education and also improve the accountability and transparency of health care delivered to patients.	-In-patients who had received pharmaceutical services on the medical wards from PharmD pharmacists within the last two weeks prior to interview. The nurses were requested to identify and invite patients who were willing to participate. Interviews took place bed-side on patients’ wards with respect for the privacy of patients’ interviews. -Out-patients at chronic disease clinics (e.g., DM clinic, oncology clinic and warfarin clinic), who had received counselling services from PharmD pharmacists within the last three months. To minimise the selection bias from pharmacists, the patients had been selected by the researcher via OPD card screening on the visit day. If patients had pharmacy counselling in the last three months, then the researcher asked the pharmacist to invite patients to participate during the lag interval, while those patients were waiting to see their doctor or waiting for their prescription. Interviews took place in private counselling rooms.	-Interview
9. General population (n=7) -Taxi driver -Merchant -Government officer - Non-governmental organisation (NGO)	0 1 1 1	0 0 0 0	1 2 0 0	0 0 1 0	1 3 2 1	-The roles of pharmacists and pharmacy education have changed. The perception of the general public regarding this change is important; for example, how they perceive the new role of pharmacists, the knowledge and skills required of pharmacists to provide better services for them, and whether they trust the advice that they may receive from pharmacists. -Their opinions will help to determine how the pharmacy profession and education can improve public understanding and perceptions of pharmacists in the future.	General population at a local supermarket in Bangkok (capital city), Ubon Ratchathani and Songkhla province (outside the capital city).	-Interview
Total	40	28	48	14	130			

^a^ *U*=University, ^b^ participants were asked for verbal consent before skype interview or telephone interview

Stakeholders who met the inclusion criteria and provided their consent for their interview data being included in a PhD thesis and publications [65] were recruited and interviewed using an interview guide (see Table 2). All participants were sent a participant information sheet explaining the study to them [59, 65]. The interview guide was developed based on both the purpose of this study and a literature review. Pilot interviews with two academic staff members, two pharmacists, one nurse, one doctor, one member of the general population and two students were conducted to refine the interview strategy. The pilot interviews were included in the data analysis. Recruitment was conducted by purposive sampling and snowball sampling. Sampling and data collection was guided by emerging themes and continued until the point of data saturation that was established when the interviews did not yield any new or emerging themes [66] and the depth and extent of data collection and data analysis seemed sufficient to allow the researcher to tell a reasonable story [67, 68]. The saturation was reached with varying numbers of participant from each sub-group of stakeholders. The depth and extent of data collection depended on participants’ roles and involvement in pharmacy education; for example, saturation was reached with 20 academic members from three specialised area (e.g. pharmaceutical care, pharmaceutical sciences and social and administration pharmacy) and different type of PharmD programmes offered (e.g. PC-PD, IP-PD), with 5 doctors and 5 nurses who have different length of experience of working with PharmD pharmacists, with 10 policy makers who had different roles from the Pharmacy Council of Thailand and the Pharmacy Education Consortium of Thailand, and with 7 students from public and private universities. However, additional 1–2 participants from each subgroup of stakeholder were interviewed to confirm the saturation of themes.

**Table 2:** Interview guide

	Questions
General/background information	Personal information: education, work experience, age, area of expertise, year of current work experience.
Feelings and attitude towards a 6-year PharmD programme	How do you feel about the all-PharmD policy in Thailand?
Impact of curriculum change	What will be the impact of the curriculum change on education, pharmacy practice and the health care system?
Policy makers, pharmacy experts and academic staff	
-Historical series or process	Please tell me about the history of PharmD curriculum development in Thailand.
-Reason for changing	Why do Thai pharmacy education have to change to a 6-year PharmD curriculum?
	Who/which other organisations support this policy and why?
	How does the PharmD curriculum value/suit the context of Thailand?
	(Probes: health care system, quality of patient care, pharmacists’ roles, law and regulation)
-Involvement	Please tell me about your role and responsibility in changing the curriculum policy.
	Please tell me about the influence or motivation (in general/for your contribution) for this change.
-Process of changing policy	What are the differences between the new PharmD programme and previous programme (e.g., BPharm, a traditional PharmD programme)?
	How will the new curriculum change?
	Manner of enacting of change: policy/real practice
	Process in which you are involved
	Readiness, preparedness for this change
	Key learning, key enablers, significant challenges/ barriers
	What do you need in terms of support?
Pharmacy students	Motivating factors to study pharmacy
	Choice of faculty of pharmacy
	Perceptions of the 6-year PharmD programme
	Experience as a final year student in the new PharmD programme
	Impact of curriculum change (on student and family/ pharmacy profession/ career image)
	Future career ambitions and views of career development
Parents	Perceptions of the 6-year PharmD programme
	Parental support for their children’s education
	Experience as a parent of a PharmD student Impact of curriculum change (on student and family/ pharmacy profession/ career image)
	Future career ambitions and views of career development
Employers	Perceptions of the 6-year PharmD programme
	General expectation of PharmD graduates
	Differences in competencies/duties between BPharm and PharmD pharmacists?
	Satisfaction with BPharm and PharmD pharmacists in terms of competencies and pharmacy services
	Expectations from pharmacists’ roles
	Expectations from pharmacy education institutions/pharmacy curriculum
	Impact of curriculum change (on student and family/ pharmacy profession/ career image)
Pharmacists	Perceptions of an all-PharmD programme
	What are the differences in terms of about competencies/duties between BPharm
	and PharmD pharmacists?
	Expectations of into the new curriculum and real work situation
	How do you perceive about PharmD pharmacists in your workplace settings?
	Are there any differences in competencies between PharmD and BPharm pharmacists?
	What makes a PharmD pharmacist different from a BPharm pharmacist?
	What is the barrier to move from the traditional pharmacy to advanced pharmacy practice?
	How to across the barrier?
	What do you expect from a PharmD pharmacist?
	Preceptor roles
	Specific questions for PharmD pharmacists
	How often/ how many days a week do you intervene regarding patients’ medications?
	How do PharmD pharmacists add value to patient care?
	How do you think your patients think about you?
	Are there any differences in competencies between PharmD and BPharm pharmacists?
Health care providers	How often do you contact a PharmD pharmacist regarding patients’ medications?
	How do you perceive PharmD pharmacists in your workplace settings?
	Are there any differences in competencies between PharmD and BPharm pharmacists?
	Are you satisfies with the pharmaceutical care services from PharmD pharmacists?
	What is the barrier to move from traditional pharmacy to advanced pharmacy practice?
	What do you want to suggest to improve PharmD services?
	Perceptions of pharmacy curriculum change to a 6-year PharmD programme
	Expectations of the new curriculum and pharmacy practices in real workplace situation
	Suggestions about multidisciplinary education
Patients/ general public	Please explain the services that you receive from your pharmacists?
	How do you think about your pharmacist? Are you satisfied with the pharmaceutical care services?
	What do you want to suggest about improving pharmacy services?
	Do you know how long the pharmacy programme is?
	How do you think that pharmacy education changes with a 6-year programme?
Closing questions	Is there anything you would like to add about an all-PharmD programme?
	Is there anything that concerns you about the interviews or that you would like to ask me before we finish?
	Would you like to check your transcript after the interview?

The interviews lasted between 45–60 min. All interviews were audio recorded with informed consent. The interviews had two sections, an introduction and personal background section (e.g., age, education, career, area of expertise, year of current work experience) and questions about their past and current experiences regarding the transition to an all-PharmD programme. Characteristics of the interviewees are given in Table 3. TC, a PhD research student has been a university lecturer in clinical pharmacy area at Ubon Ratchathani University, Thailand, for the last ten years and has observed the changes in pharmacy education; she has not been involved in influencing the policy of the all-PharmD programme. TC interviewed the participants in the setting of their choice (usually their workplace). 124 interviews were conducted face-to-face, five via telephone, and one was a Skype interview. PY and SM are university associate professors in Thailand. PY had been involved the transition period as a deputy dean of an academic affair and now is a policy maker of the PCT. SM has not been involved in influencing the policy of the all-PharmD programme. BL and CA are academic supervisors of TC who has not been involved in the transition of an all-PharmD programme in Thailand.

**Table 3:** Descriptive characteristics of participants (*n* = 130)

Participants	Policy makers	Pharmacy Experts	Educators	Pharmacy students	Parents	Physicians	Nurses	Pharmacists	Pharmacy technicians	Patients	General public
Number of participants	10	13	25	9	4	7	6	30	5	14	7
Gender											
Male	7	7	13	4	1	5	0	7	1	4	4
Female	3	6	12	5	3	2	6	23	4	10	3
Age group (years)											
20-30	0	0	3	9	0	0	2	8	3	2	0
31-40	0	0	2	0	0	3	1	11	1	2	2
41-50	0	6	13	0	2	1	2	6	1	5	4
51-60	5	6	6	0	2	3	1	5	0	2	1
More than 60	5	1	1	0	0	0	0	0	0	3	0
Work experience (years)											
less than 5	0	0	3	0	N/A	0	2	1	3	N/A	N/A
5-10	0	0	0	0	N/A	2	0	15	1	N/A	N/A
11-15	0	0	0	0	N/A	1	0	2	0	N/A	N/A
16-20	0	2	8	0	N/A	0	1	4	1	N/A	N/A
more than 20	10	11	14	0	N/A	4	3	8	0	N/A	N/A
Working area (years)											
-Hospital pharmacy (tertiary care hospital, public)	1	3	0	0	0	4	3	11	3	0	0
-Hospital pharmacy (community hospital, public)	0	1	0	0	0	3	3	4	2	0	0
-Hospital pharmacy (private)	0	1	0	0	0	0	0	2	0	0	0
-Community pharmacy	0	3	0	0	0	0	0	5	0	0	0
-Industrial pharmacy	1	2	0	0	0	0	0	2	0	0	0
-Public health and consumer protection	1	2	0	0	0	0	0	2	0	0	0
-Marketing pharmacy	0	1	0	0	0	0	0	2	0	0	0
-Research and development	0	0	0	0	0	0	0	2	0	0	0
-Academic (pharmaceutical care)	3	0	10	0	0	0	0	0	0	0	0
-Academic (Pharmaceutical technology	4	0	10	0	0	0	0	0	0	0	0
-Academic (Social and administrative pharmacy)	0	0	5	0	0	0	0	0	0	0	0
Highest education											
1. Physician											
-Doctor of Medicine (MD)	0	0	0	0	0	1	0	0	0	0	0
-MD, Diploma	0	0	0	0	0	2	0	0	0	0	0
-MD, Specialist	0	0	0	0	0	4	0	0	0	0	0
2. Nurse											
-Bachelor of Nursing Science	0	0	0	0	0	0	4	0	0	0	0
-Master of Nursing Science	0	0	0	0	0	0	2	0	0	0	0
3. Pharmacist											
-BPharm	0	2	0	0	0	0	0	11	0	0	0
-MPharm/ MSc	2	7	2	0	0	0	0	12	0	0	0
-PharmD	0	0	0	0	0	0	0	7	0	0	0
-PhD	8	3	17	0	0	0	0	0	0	0	0
-PharmD, Pharmacy residency	0	0	3	0	0	0	0	0	0	0	0
-PharmD, Board specialties	0	1	3	0	0	0	0	0	0	0	0
4. Pharmacy technician											
-Diploma of public health (Pharmacy Technique)	0	0	0	0	0	0	0	0	4	0	0
-Bachelor	0	0	0	0	0	0	0	0	1	0	0
5. Patient, parent, public											
-Less than secondary education	0	0	0	0	0	0	0	0	0	5	1
-Secondary education/ vocational education	0	0	0	9	2	0	0	0	0	5	2
-Bachelor	0	0	0	0	2	0	0	0	0	4	3
-Higher than bachelor	0	0	0	0	0	0	0	0	0	0	1

Audio recordings were transcribed verbatim in Thai and were checked twice for accuracy with the recordings by TC and one Thai pharmacist [69]. Transcripts were sent back to the interviewees for them to read or modify the transcript if necessary [59, 70]. However, the majority of participants chose not to check the transcripts. The transcripts were checked and confirmed the correctness by ten requested interviewees [62]. Thai transcripts were translated to English to comply with the required audit trail [71] by a Thai researcher (TC), who is a PhD student in the UK. The audit trail is an essential part of rigorous qualitative study that will able to track how the data were analysed and how themes were generated through interviews and interpreted to assess the trustworthiness of the research [72]. Additionally, it was essential that the transcripts be translated to English as CA, a non-Thai researcher and a senior qualitative researcher was directly involved in the analysis and coding processes [73]. Meaning-based translation [74] from Thai to English was performed by TC and had forward-blind backward translations process [74] to check the correctness of the translation. Twenty English transcripts were checked against the Thai transcripts by TC and a bilingual Thai-English pharmacy academic researcher. Two translators reached consensus regarding the English translation. Then, convenience blind backward translations [74, 75] of English transcripts into Thai were undertaken for 13 of the 130 transcripts (10 %) by the fluent Thai-English bilingual speaker. This process was performed to validate the translations and ensure no loss of conceptual equivalence had taken place [76].

English transcripts were then analysed thematically using NVivo qualitative data analysis software (QSR International Pty Ltd., Version 10, 2012) [77]. This study used an inductive (data-driven with themes emerging directly from the data) thematic analysis approach [78] and was influenced by the principle of grounded theory [59, 66, 79] (e.g., the general explanation or theory development generated or “grounded” in data from the views of participants who have experience with the process; while the researcher collects data, they begin analysis and go back to the field to collect more information until data saturation is achieved, used constant comparison analysis and theoretical sampling to maximise the variation of participants) [62, 64]. However, this study aimed to understand and develop an explanation regarding this transition, which differs from pure grounded theory as grounded theory aims to build the theory [79].

**Fig. 2:**
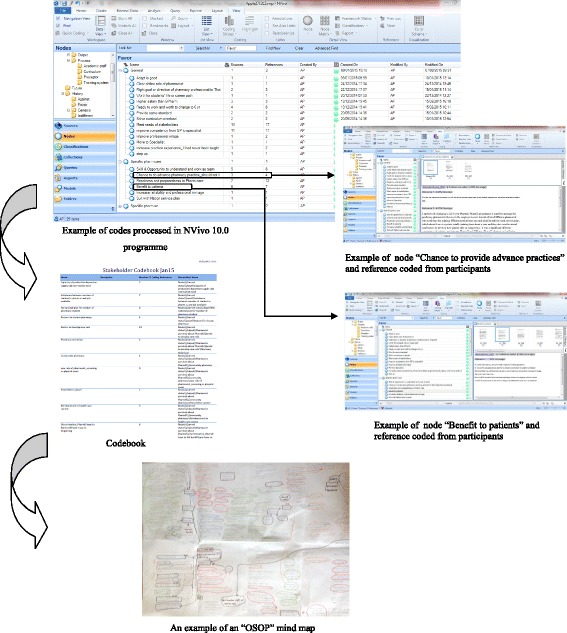
The process of theme development with the “One Sheet of Paper, OSOP” method

The data analysis was undertaken with the following steps [80]:

1. The analysis began after the first two interviews for each stakeholder group were transcribed and continued during and after data collection.
2. The first two English transcripts from each stakeholder group were read and re-read to gain an understanding of the interviewees’ perceptions and experiences [59, 78] by two researchers (TC, CA). They independently read transcripts carefully line-by-line, noted possible codes within the transcript hard copy, and then started to code and produce a coding structure.
3. The coding structure was revised and further developed [59]. The codes were compared and discussed by two authors (TC, CA) [62, 81–85].
4. The coding process was started again for all transcripts using the NVivo 10.0 software.
5. A constant comparative analysis (moving back and forth between the identification of similarities and differences among emerging categories) approach was taken; for example, new information that might add to the code was constantly compared with previous codes or categories or themes that it might fit, or it was determined whether a new code or category should be created. The emerging themes were coded and constantly compared and contrasted with other interviewees’ transcripts [86].
6. To establish the reliability or the stability of responses to multiple coders of the dataset, this study used the inter-coder agreement process. A draft code book was developed. It was aimed at determining the agreement of the coding in terms of code names and coded passages. TC and CA independently coded another two transcripts using a codebook and comparing codes. They considered the agreement of coding for these passages to be more than 80 % of coding [62].
7. TC read through all transcripts repeatedly and coded them for analysis while a PhD supervisor (CA) checked and revised the coded text [73].
8. Thematic analysis was carried out using the “One Sheet of Paper, OSOP” mind map method to ensure that all the codes extracted within each theme were included and compared in the analysis [59]. This process aimed to find a story in the data, which involved reading through each section of the data and making notes on a single sheet of paper, and was used to ensure that all different issues were raised by the coded extracts, along with the relevant participant IDs [59, 87] (see Fig. 2). TA, CA, and BY considered the development of broader themes from the codes.

Transcript coding (the process by which a qualitative analyst links specific codes to specific data segments or the process of organising data) involved four steps as follows:

1. In vivo coding or initial coding in-text indicates the coding of special terms of participants. In vivo codes help us to preserve interviewees’ meaning regarding their perspectives or experiences in the coding itself [67]
2. Open coding (concepts/categories or free codes/free nodes in NVivo 10.0 software)
3. Axial coding (relationship between categories or tree code or tree nodes in NVivo 10.0 software)
4. Selective coding (core categories or higher level of concepts/themes) [83, 88]

This study took the preliminary analyses consisting of themes back to six participants (e.g., two policy makers, two academic staff members, experts from the consumer protection area and industrial pharmacy), who would share their views of the analyses [62] to triangulate the data. They agreed on the accuracy and credibility of the findings. The findings and data from different sources [61–63] (e.g., government document, conference proceedings, unpublished reports, unpublished meeting minutes) were compared and contrasted; facts provided by the participants were confirmed.

## Results

The findings revealed three major themes regarding stakeholders’ perceptions towards the transition to the 6-year PharmD programme in Thailand: influences of an all-PharmD programme in Thailand, perceived benefits and concerns regarding the transition to an all-PharmD programme.

### Theme 1: Influences of an all-PharmD policy **4**

**Fig. 3:**
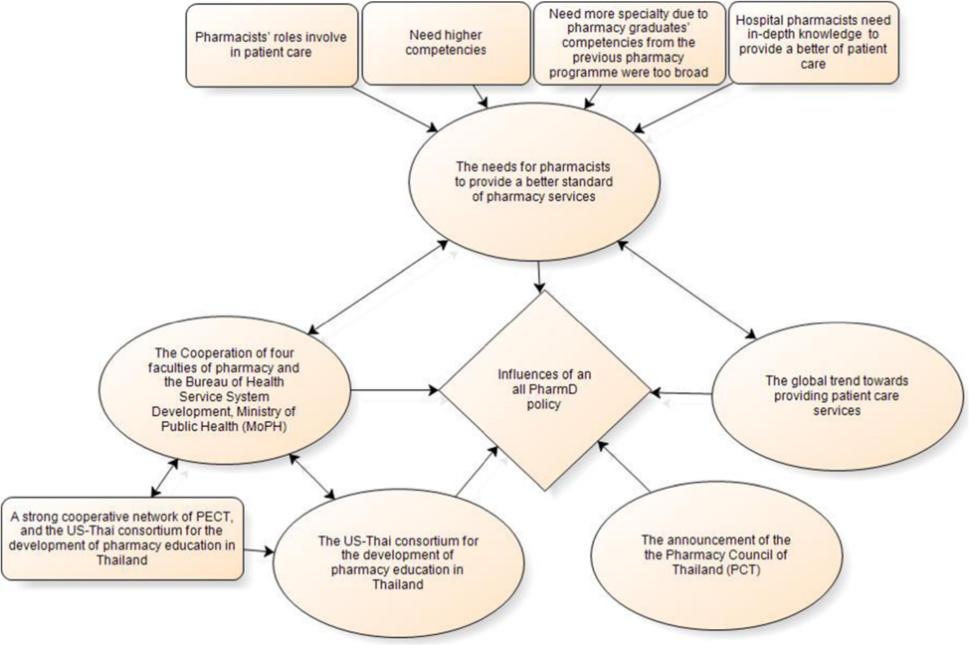
Theme “Influences of an all-PharmD policy”

The most frequent influence of an all- PharmD policy noted in the findings was the need for pharmacists to provide a better standard of patient care due to the competencies of pharmacy graduates from the previous pharmacy programme (a 5-year BPharm) being too broad and not being suitable for pharmacy practice in the patient care area in Thailand. The detailed themes and subthemes are presented in Fig. 3. The subthemes and supporting quotes are presented in Table 4.

*“Hospital pharmacists should have the in-depth knowledge to serve patients by doing more than dispensing medication, so a PharmD in pharmaceutical care is the answer.” (Policy maker 9)*

**Table 4:** Subthemes and supporting quotes within the theme “Influences of an all- PharmD policy”

Key theme: Influences of an all- PharmD policy			
Subthemes	Concepts	Participants^a^	Example of supporting quotes
The needs for pharmacists to provide a better standard of pharmacy services	Hospital pharmacists need in-depth knowledge to provide a better of patient care	PM9	*“Hospital pharmacists should have the in-depth knowledge to serve patients by doing more than dispensing medication, so a PharmD in pharmaceutical care is the answer.” (PM 9)*
	Need more specialty due to pharmacy graduates’ competencies from the previous pharmacy programme (a 5-year BPharm) were too broad	AC1_PSc_U1, AC14_SAP_U5, AC22_D_U5, PM1, PM3, PM5, PM8	*“Policy makers believed that separate pharmacy specialty by have separate curriculum and training will meet the social need rather than general pharmacist.” (AC14_SAP_U5)*
			*“They see the problem that our pharmacist was too generalised. Employers thought graduates did not have in-depth knowledge either in patient care or product focused.” (AC22_D_U5)*
			*“Another reason was regard to the 5-yearr programme which does not make us experts in medicines and patient care.” (PM1)*
	Need higher competencies	AC3_PC_U4, AC14_SAP_U5, PM1, PM8	*“The reason is increasing competency which should have to separate to two special tracts (PC-PD, IP-PD) with more practice training.” (AC14_SAP_U5)*
	Pharmacists’ roles involve in patient care	AC8_PC_U4, PM1	*“Now pharmacist’s roles are more involvement in patient care.” (AC8_PC_U4)*
The global trend towards providing patient care services	The global trend of patient care services	PM1, PM7, PM8	*“We considered the direction at an international level and saw that the trend for developed countries was normally to adapt a practice role that emphasised patient care. This might be more suitable due to the PharmD programme giving us higher competencies as pharmacists.” (PM1)*
			*“Global trends are likely to come to specifically in pharmaceutical care. This global trend needs a quality pharmacy the other way around, one more heavily weighted towards pharmaceutical care. Pharm care was requiring more profound training for new pharmacists to be ready to work.” (PM7)*
The Cooperation of four faculties of pharmacy and the Bureau of Health Service System Development, Ministry of Public Health (MoPH)	The Cooperation of four faculties of pharmacy and the Bureau of Health Service System Development, Ministry of Public Health (MoPH)	PM1, PM9	*“There were agreed and jointed investment between four faculties of pharmacy and the Bureau of Health Service System Development, Ministry of Public Health (MoPH). It was an atmosphere of new learning.” (PM1)*
The US-Thai consortium for the development of pharmacy education in Thailand	A strong cooperative network of PECT, and the US-Thai consortium for the development of pharmacy education in Thailand	AC3_PC_U4, AC4_PC_U3, AC10_PSc_U5, AC21_D_U5, AC22_D_U1, EXP13_HOS, PM1, PM2, PM10	*“The administrator of faculty of pharmacy had been visited the pharmacy school in the US. They thought that they should have a clinical pharmacy focused programme which this future direction of pharmacist.” (*AC4_PC_U3*)*
			*“The PECT and Ministry of Public health were co-ordinate for US-Thai consortium. We received idea from US and learn from them. Both academic and pharmacists were train from US.” (AC10_PSc_U5)*
			*“A 6-year PharmD programme was implemented by policy of the pharmacy council. Literally, I think it was influenced from US-Thai consortium which they (pharmacy council) had accepted concept from US quite a lot.” (AC21_D_U5)*
			*“It started from PECT since 1996–1997 that we have Thai-US consortium which is a subset of the project of pharmacy workforce development of MoPH.* *PECT thought that change to a 6- year programme might be the way to develop the pharmacy profession in Thailand as in a developed country.” (PM2)*
			*“The result of the consortium has had a great impact on Thai pharmacy education and the Thai pharmacy profession.” (PM10)*
The announcement of the the Pharmacy Council of Thailand (PCT)	The announcement of the the Pharmacy Council of Thailand (PCT)	AC21_D_U5, PM2, PM3, EXP12_COM, EXP13_HOS	*“The Pharmacy Council stated that if we did not provide a 6-year PharmD curriculum, our students will not qualify to take the licensure examination. All faculties have to adapt their programmes to 6 years, no matter whether it is pharmaceutical sciences or pharmaceutical care.” (AC21_D_U5)*
			*“This policy was commenced as law by the Pharmacy Council.” (PM2)*
			*“If PCT did not announce as a regulation, this would never be ready, right? It was very confused at that time. We needed someone to make decisions for our future.” (PM3)*
			*“The intention of the PCT was good. They proposed to clarify the position of the pharmacy profession which might useful to our society and our profession.” (EXP 13_HOS)*

^a^Abbreviation

*AC_PSc* Academic member in pharmaceutical sciences area, *AC_PC* Academic member in pharmaceutical care area, *AC_SAP* Academic member in social and administrative pharmacy), *AC_D* Dean, *EXP_HOS* Expert in hospital pharmacy, *EXP_COM* community pharmacy, *EXP_PSc* Expert in industrial pharmacy, *EXP_PB* Expert in public health and consumer protection, *EXP_MP* Expert in marketing pharmacy, *N* Nurse, *PH_HOS* Hospital pharmacist, *PH_COM* community pharmacist, *PH_IP* Industrial pharmacist, *PH_PB* Pharmacist in public health and consumer protection, *PH_MP* Pharmacist in marketing pharmacy, *PH_RD* Pharmacist in Research and development, *PHY* Physician, *PM* Policy maker, *PTECH* Pharmacy technician, *PT_IPD* Patient in In-Patient Department, *PT_OPD* Patient in Out-Patient Department, *PUB* Public/general population, *ST* Student, *PR* Parent, *U1* University1, *U2* University2, *U3* University3, *U4* University4, *U5* University5

Policy makers who were involved in this transition remembered the initiatives of this change as part of a global trend towards providing patient care services.

*“We considered the direction at an international level and saw that the trend for developed countries was normally to adapt a practice role that emphasised patient care. This might be more suitable due to the PharmD programme giving us higher competencies as pharmacists.” (Policy maker 1)*

A factor that has also been mentioned as an influence on the transition to an all-PharmD programme was the Cooperation of four faculties of pharmacy and the Bureau of Health Service System Development, Ministry of Public Health (MoPH), in the development and establishment of a master’s degree in clinical pharmacy programme via a modular system. This foundation of clinical pharmacy activities in real workplace settings was supported by the US-Thai consortium for the development of pharmacy education in Thailand (founded in May 1994 by the Pharmacy Education Consortium of Thailand, PECT).

*“The result of the consortium has had a great impact on Thai pharmacy education and the Thai pharmacy profession.” (Policy maker 10)*

The mission of this consortium was to provide Thai pharmacy academic staff and pharmacists, who were selected by a Royal Thai Government Panel, to access advanced professional (PharmD) or graduate (PhD) studies and training in selected pharmacy schools in the US. Pharmacy educators adopted the US PharmD programme to establish the first Thai PharmD programme (pharmaceutical care) at the Faculty of Pharmaceutical Sciences, Naresuan University in 1999.

However, the big drive came from the announcement of the Pharmacy Council of Thailand (PCT) in 2008 that, starting in 2014, all new pharmacy graduates would have to graduate from pharmacy faculties accredited by the Council through the 6-year PharmD curriculum only.

*“The Pharmacy Council stated that if we did not provide a 6-year PharmD curriculum, our students will not qualify to take the licensure examination. All faculties have to adapt their curriculum for all their programmes to 6 years.” (Academic member 21)*

### Theme 2: Perceived benefits of the transition of pharmacy education to an all-PharmD programme

**Fig. 4:**
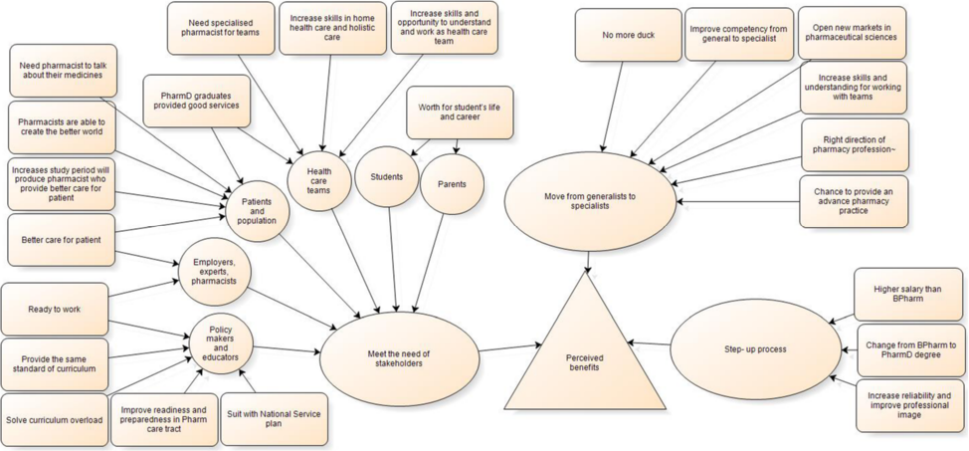
Theme “Perceived benefits”

**Table 5:** Subthemes and supporting quotes within the theme “Perceived benefits”

Key theme: Perceived benefits			
Subthemes	Concepts	Participants^a^	Example of supporting quotes
Step- up process	Change from BPharm to PharmD degree	AC4_PC_U3, AC19_PC_U3	*“It was a step up from the Bachelor degree to the professional degree which is a 6-year professional degree similar to other health professions such as physicians and dentistry.”(AC 4_PC_U3)*
		AC20_PC_U3	
	Increase reliability and improve professional image	AC_D_U3,	*“This change might increase reliability to pharmacy professional.”(PH2_HOS)*
		PH2_HOS, PH23_PB, PH21_IP, PH21_IP, PH13_MP, PH14_RD, PM1	*“We should give the good services first so we will get trust from public.” (PM1)*
	Higher salary than BPharm	EXP9_HOS, EXP10_HOS, PH24_HOS, PH14_COM, PH18_COM, PR2, ST9	*“The career progression of PharmD pharmacist is faster than a 5 year programme. The 6-year PharmD graduates have higher salary than a 5 year pharmacist who works for 3–4 year before PharmD graduates.” (EXP10_HOS)*
			*“Career ladder is also good because a 6 year graduates had salary which equivalence to a Master degree. Duration to higher position is shorter than a 5 year BPharm.” (PH24_HOS)*
Move from generalists to specialists	Chance to provide an advance pharmacy practice	AC22_D_U2, AC25_U3, EXP9_HOS, PH20_IP, PH25_HOS, PM1	*“A 5-year BPharm programme does not make us experts either in medicines or patient care. This 6-year programme would give us higher competencies.” (PM 1)*
	Increase skills and understanding for working with teams	AC9_SAP_U1, N1, PH2_HOS, PH25_HOS, PHY5	*“PharmD graduates had better skills in patient care. They had more confident and work well with health care team.” (PH2_HOS)*
	Clear definition of pharmacists’ roles	EXP13_HOS, PM1, PH2_HOS, ST8	*“The intention of PCT was good. They proposed to clarify the position of the pharmacy profession which might useful to our society and our profession.” (EXP13_HOS)*
			*“We see the clearer picture of patient care. It is a view, that this is the direction we are going to do and the old curriculum was still missing a lot of thing that we need to learn.” (PM1)*
			*“Pharmacy professional will be looked clearer from this pharmaceutical care.” (PH2_HOS)*
	Improve competency from general to specialist	AC3_PC_U4, AC21_D_U5, AC22_D_U1, EXP2_COM, EXP9_HOS, EXP10_HOS, EXP11_MP EXP13_HOS, PH24_HOS, PHY1, PHY4, PM1, PM2, PR1, ST1, ST6, ST7	*“Pharmacist should prepare for specialty as well so they can work as a health care team and easy to communicate.” (PHY4)*
	Right direction of pharmacy profession	EXP9_HOS, PH23_PB, PH24_HOS, EXP13_HOS, AC13_PC_U1	*“It is the right way and it was partial success. I give credit to everyone. Everyone has good intention but it will be sustained or not.” (AC13_PC_U1)*
	No more duck	PH19_RD, PH22_HOS, PM1, ST3, ST4, ST5	*“A 5-year BPharm programme does not make us experts either in medicinal products or patient care. This 6-year programme would give us higher competencies.” (PM1)*
			*“We are too broad, like a duck. We should good at something better than know everything, but we are not good in anything.” (ST3)*
	Open new markets in pharmaceutical sciences	AC17_SAP_U1, PH29_COM, PH30_COM, PM5, PM8	*“Job markets are still available but we have to open new markets, such as areas in vaccines, blood substitutes, nanotechnology, in herbal medicine. Faculty should move to design our graduates.” (PM5)*
Meet the need of stakeholders	Patients and population: better care for patient	AC3_PC_U4, AC5_SAP_U1, N1, N2 PHY5, PHY7, PH2_HOS, PH15_COM, PH16_COM, PH14_MP, PM9, PT_IPD4, PT_OPD2, PT_OPD6, PT_TECH1, PT_TECH2, PUB2, PUB5	*“PharmD graduates have better skills in patient care. They are more confident and work well with health care teams.” (PH2_HOS)*
			*“Employers from hospitals and community pharmacies had high satisfaction with PharmD graduates.” (PM9)*
			*“For the 6-year Pharmacy programme, I think it must be good. I know that if patients have problems with medicine they can talk with* *a pharmacist and the pharmacist will talk to the doctor for them. It makes for better care for us.” (PT_OPD2)*
	Patients and population: need pharmacist to talk about their medicines	PT_IPD2, PT_IPD4, PT_TECH4, PT_TECH5	*“Pharmacist told us about how many tablets we have to take or what the medicines used for. But I think it is not enough. They should tell us more about how to monitor other symptom s which might happen or how to monitor about side effect.” (PT_IPD4)*
	Patients and population: pharmacists are able to create the better world	PUB7, PUB3	*“I am glad to know that some faculties encouraged their pharmacy students to visit homes in poor community and help people about drug related problems. Students should know about suffering in people’s life who did not know their right to accessible to health care system.* *Pharmacy professional had impact on society and pharmacists are able to create the better world.” (PUB7)*
	Patients and population & Health care teams: PharmD graduates provided good services	EXP2_COM, N3, N5, N6, PHY2, PHY6, PT_IPD1, PT_IPD3, PT_IPD4, PT_IPD5, PT_OPD1, PT_OPD2, PT_OPD3, PT_OPD4, PT_OPD5, PT_OPD6, PT_OPD7, PT_OPD8, PT_OPD9, PT_TECH3, PUB1, PUB3, PUB4	*“The 6-year pharmacists help us to adjusted doses, increase patients’ compliance and follow-up drugs ‘side effects.” (PHY2)*
			*“I think it is good to have a pharmacist on ward. We discuss and shared opinions which is beneficial for our patients.” (PHY6)*
			*“They (PharmD graduates) explained very well. They told me about diet, exercise and medicines.” (PT_OPD1)*
			*“Her (PharmD graduates) service is very good. She takes a good care to patients. I feel courage and able to talk with her. She makes us feel warm. She is willing to treat us. I feel confident in her knowledge for 100 percent sure.” (PT_OPD2)*
			*“Pharmacist taught me how to use this medicine. I have to remove it from the sealed pouch, peel off this, and clean the skin that the patch will be applied. Pharmacist also told me that this medicine will have side effect.”(PT_OPD5)*
			*“The 6 year graduates are very important team member especially in medicinal wards and ICU.” (N3)*
Meet the need of stakeholders (continued)	Employers: ready to work	AC6_SAP_U4, EXP3_HOS, EXP9_HOS, EXP10_HOS, EXP8_PB, PH2_HOS, PH10_HOS, PH24_HOS, PH25_HOS, PH16_COM	*“PharmD graduates are ready to work. It fulfil the need of employers” (AC6)*
			*“The 6-year PharmD programme is a perfect package. There was a significant different in the competency of patient care between PharmD and BPharm. I will choose a six year programme graduate because they are ready to use.” (EXP9_HOS)*
			*“PharmD graduates have the skills to approach other health care providers. They are ready to work after graduation.” (EXP10_HOS)*
	Policy makers and educators: solve curriculum overload	AC2_PC_U4, PH2_HOS, PM5	*“I think the 6- year PharmD programme is good because I graduated from a 5-year programme which was too tight and clerkship period is too short.” (PH2_HOS)*
			*“The previous programme had curriculum overload but still lack of skill. Policy makers discussed about this issue that if we change to a 6 year curriculum, this will provide more time to practice.” (PM5)*
	Students and parents: worth for student’ life and career	PH25_HOS, PR1, PR3, PR4, ST2	*“I think it is worth another year. It is an opportunity to choose the way of life from the internship experience. Learning a six year programme made the longer internship and had much more experiences.” (PH25_HOS)*
			*“The 6-year programme provides more practice and includes additional things to learn.” (PR1)*
			*“I think the-6 year programme is worth. We can expose the real situation. In the sixth year we had clerkship all year. It is very useful that we can learn a lot from training.”* (ST 2)
	Patients and population: increases study period will produce pharmacist who provide better care for patient	PHY5, PH26_HOS, PM8, PTECH3, PT_IPD4, PT_IPD5, PT_OPD6, PUB6	*“The time for practicing in previous programme was too short and had too much type of training sites.” (PM8)*
			*Contrast view*
			*“I think all pharmacists have high competence. There was no different between a 5-year and 6-year graduates. They did not have a different job description. They are working together.” (PTECH3)*
	Policy makers and educators: improve readiness and preparedness in Pharm care tract	AC1_PSc_U1, AC2_PC_U4, EXP9_HOS, EXP13_HOS, PH9_HOS, PH25_HOS	*“It will be very useful toward preparing the student for their work. A 5-year programme provided only 3 months of training rotation. They have not enough time to practice.” (EXP 13_HOS)*
			*“PharmD graduates had better skills in patient care. They had more confident and work well with health care team.”* (PH9_HOS)
Meet the need of stakeholders (continued)	Health care teams: Increase skills and opportunity to understand and work as health care team	AC4_PC_U4, AC18_PC_U3, AC19_PC_U3, EXP11_MR, N1, PH10_HOS, PH25_HOS, PHY5, PTECH1, EXP11_MP	*“The 6-year pharmacy graduates have higher knowledge. Their views or critical thinking seem wider or have more understanding about the concept of a multidisciplinary team. We are friends who will walk together.” (N1)*
			*“We extend to six year in order to do more work with patient. We had the opportunity to work with doctors and nurses. When we work with team, I felt that I am valuable. Dispensing is limiting our potential. We used fully potential when we work on ward.”(PH25_HOS)*
			*“We work as health care team. Doctors cannot work by themselves. The processes need other profession to take care and check. Pharmacists help physicians about medicines. That is a confirmation process for the best care to our patients.” (PHY5)*
			*“The six-year graduates have more in-depth knowledge.” (PTECH1)*
	Policy makers and educators: provide the same standard of curriculum	AC22_D_U1, EXP13, PM3, PM8	*“At that time our country had both 5-year programme and 6-year programme that might have some confusion in term of the difference of certified of degree and salary of graduates.” (AC22_D_U1)*
			*“They all have to be graduates of the six-year programme and managed in a similar manner.” (PM 3)*
	Health care teams: increase skills in home health care and holistic care	EXP12_COM, PH4_HOS, PH5_HOS, PH25_HOS, PH26_HOS, PH27_HOS, PHY3	*The six-year graduates provide home health care service and follow up patient at home. They work like a family medicine but they are family pharmacist. They had been added this skill. In the past, they manage only medicine, but now they provide holistic care to patients. (PHY3)*
	Health care teams: need specialised pharmacist for teams	EXP3_HOS, EXP8_PB, PH11_HOS, PH28_HOS, PHY4, N4, N6	*“Pharmacist should prepare for specialty as well so they can work as a health care team and easy to communicate.”* (PHY4)
	Policy makers and educators: suit with National service plan	AC3_PC_U4, EXP9_HOS, PH7_HOS, PH8_HOS, PH12_HOS	*“The 6-year programme follows context of national policy towards health care and services excellence in Asia pacific. The National service plan also encourage the developing of pharmacy services in all level of care.” (AC3_PC_U4)*

^a^Abbreviation

*AC_PSc* Academic member in pharmaceutical sciences area, *AC_PC* Academic member in pharmaceutical care area, *AC_SAP* Academic member in social and administrative pharmacy), AC_D Dean, *EXP_HOS* Expert in hospital pharmacy, *EXP_COM* community pharmacy, *EXP_PSc* Expert in industrial pharmacy, *EXP_PB* Expert in public health and consumer protection, *EXP_MP* Expert in marketing pharmacy, *N* Nurse, *PH_HOS* Hospital pharmacist, *PH_COM* community pharmacist, *PH_IP* Industrial pharmacist, *PH_PB* Pharmacist in public health and consumer protection, *PH_MP* Pharmacist in marketing pharmacy, *PH_RD* Pharmacist in Research and development, *PHY* Physician, *PM* Policy maker, *PTECH* Pharmacy technician, *PT_IPD* Patient in In-Patient Department, *PT_OPD* Patient in Out-Patient Department, *PUB* Public/general population, *ST* Student, *PR* Parent, *U1* University1, *U2* University2, *U3* University3, *U4* University4, *U5* University5

Most interviewees from academic institutions and pharmacy practitioners perceived benefits of the transition to an all-PharmD programme because it was a step-up process for pharmacy education and the profession and because it enhanced a move from generalists to specialists, while graduates’ users (e.g., patients, health care teams and employers) perceived benefits as the PharmD graduates would have a higher competency to meet their needs. The detailed themes and subthemes are presented in Fig. 4. The subthemes and supporting quotes are presented in Table 5.

### Step-up process

Thai pharmacy education has changed from the 5-year BPharm degree (with three main tracks: PC, PSc, SAP) and the 6-year PharmD degree focused on patient care to a single national PharmD programme (offering both pharmaceutical care (PC-PD) and industrial pharmacy (IP-PD). Faculty members thought that this change was a step up for the pharmacy profession and for pharmacy education in Thailand.

*“It was a step up from the Bachelor degree to the professional degree, which is a 6-year professional degree similar to other health professions such as physicians and dentistry.” (Academic member 4)*

Unfortunately the Thai government originally gave the PharmD the same status as a bachelor degree due to it being an entry level programme, but they later rewarded progression by matching the promotions and salaries of PharmD graduates similar to those with a master’s degree. Other 6-year programmes that have also been awarded similar to a master’s degree include the Doctor of Medicine (MD), Doctor of Dental Surgery (DDS) and Doctor of Veterinary Medicine (DVM) in Thailand.

### Move from generalists to specialists

The direction of pharmacy education in Thailand was not clear before the transition process. After the pharmacy council announced the all-PharmD policy, there were efforts to equip existing pharmacists with specialised competencies. Most interviewees thought that the transition to the 6-year programme would improve pharmacy competencies from generalists to advance general pharmacists or specialists. The frequent phrase noted in the findings was *“the Thai pharmacist should not be a****duck****anymore”*. The Thai meaning of duck is that this bird is able to perform many tasks (e.g., flying, running and swimming) but does not excel in any of them.

*“We are too broad, like a duck. We should be good at something better than knowing everything, but we are not good in anything.” (PharmD Student 3)*

*“A 5-year BPharm programme does not make us experts either in medicinal products or patient care. This 6-year programme would give us higher competencies.”* (Policy maker 1)

However, some experts and academic members perceived that the specialisation in the 6-year PharmD graduates might suit only in patient care area rather than other areas (e.g., industrial pharmacy, pharmacy marketing).

### Meet the needs of the stakeholders

The minimum credit requirement for the 5-year BPharm programme was 150, whereas the 6-year PharmD programme requires a minimum of 220 credits. The 6-year PharmD programme requires 2,000 h of practice training, which is 1,500 h more compared to the 5-year BPharm programme. The increased credits in the 6-year PharmD curriculum provide in-depth knowledge within special tracks. The one-year extension provides the PharmD students with more practice rotations. The stakeholders expect the PharmD graduates to have higher competencies and be ready to work as pharmacists due to the greater number of didactic credits and the longer training experience.

*“The 6-year programme provides more practice and includes additional things to learn.” (Parent 1)*

The majority of pharmacists who work in pharmaceutical care areas perceived the benefits of the PharmD programme, such as preparing PharmD graduates for work immediately after graduation, understanding other health care professionals, and providing high quality patient care.

*“PharmD graduates have the skills to approach other health care providers. They are ready to work after graduation.” (Pharmacy expert 10)*

*“PharmD graduates have better skills in patient care. They are more confident and work well with health care teams.” (Pharmacist 2, hospital pharmacist)*

Physicians and nurses also have positive perceptions towards the 6-year PharmD graduates’ services and thought that they were effective members of the multidisciplinary team.

*“I think it is good to have a pharmacist on ward. We discuss and share opinions, which are beneficial for our patients.” (Physician 6)*

*“The 6-year pharmacy graduates have higher knowledge. Their views or their critical thinking seem wider or have more understanding about the concept of a multidisciplinary team.” (Nurse 1)*

Patients also perceived the benefits of pharmacists in the health care team, which would provide better care for them.

*“For the 6-year Pharmacy programme, I think it must be good. I know that if patients have problems with medicine they can talk with a pharmacist and the pharmacist will talk to the doctor for them. It makes for better care for us.” (Patient 2, Out-patient Department)*

Most policy makers agreed that the transition to a single PharmD programme provided the same curriculum standard throughout the country.

*“They all have to be graduates of the six-year programme and managed in a similar manner.” (Policy maker 3)*

### Theme 3: Concerns

**Fig. 5:**
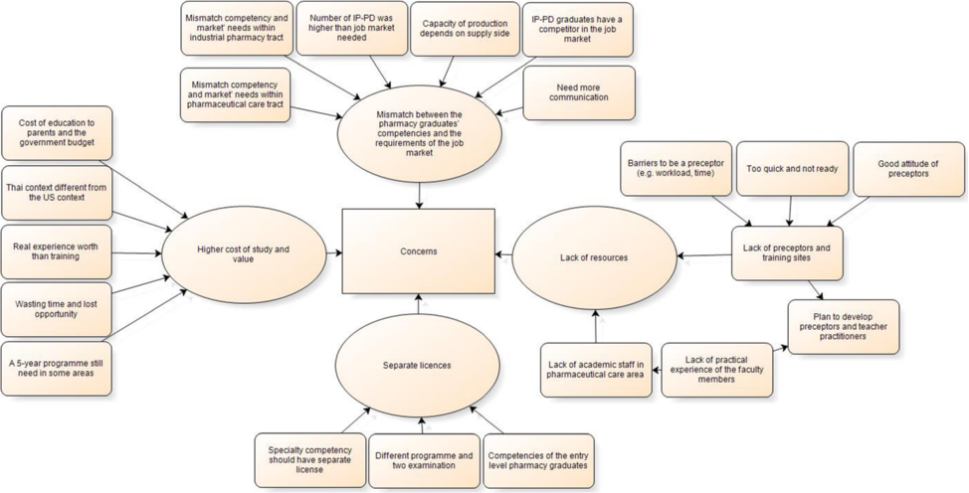
Theme “Concerns”

Interviewees such as hospital pharmacists, other health care providers and patients, students and parents, and academic staff in patient care areas were positive overall regarding the all-PharmD programme, while academic staff in the pharmaceutical sciences area and industrial pharmacy experts still had concerns about the curriculum change and suggestions for its improvement. The detailed themes and subthemes are presented in Fig. 5. The subthemes and supporting quotes are presented in Table 6.

**Table 6:** Subthemes and supporting quotes within the theme “Concerns”

Key theme: Concerns			
Subthemes	Concepts	Participants^a^	Example of supporting quotes
Higher cost of study and value	Cost of education to parents and the government budget	AC12_SAP_U4, EXP4_PSc, PR1, PR3, ST1	*“The negative effect is the cost of education to parents and the government budget of at least six hundred million baht (£12,253,000 or $18,421,000) a year, without being able to see the benefit in the short or long term. Improving pharmacists’ competencies is a great thing but we have many ways to improve them, such as, via studying or working in real life situations.” (AC12_SAP_U4)*
			*“A 5 yr programme will not increase cost. This increasing cost gave a burden to the government, students and users or employers due to when we had longer period of study, we will ask higher rate of salary in both public and private sector.” (EXP4_PSc)*
			*“I think the cost is quite heavy. In case where we do not have money, this might be a problem.” (PR 1)*
			*“The cost is not much harder because he (her son) helps himself. He got a scholarship from the faculty and works part-time job with his teacher. When he comes back home, he rarely asks for money. He needs only approximately 5,000 baht (£100 or $150) per month. He is a very good saver. He rarely buys new clothes.” (PR 3)*
			*“The Pharmacy Council should prepare salary or compensation for our degree. I heard that they increased our salary by only 1,000 baht (£20 or $31) a month compared to the 5 year programme. It is not worth it to study another one year, when we have to spend approximately 100,000 baht (£2042 or $3070) for this extended year. There should be other ways to encourage us to work hard and deserve our salary.” (ST1)*
			*ᅟ*
	Real experience worth than training	AC2_PC_U4, AC7_PSc_U4, AC12_SAP_U4	*“I think about the difference between a 6* ^*th*^ *year pharmacy student who has clerkship with an unprepared preceptor for one year and a 5- year pharmacist who works for one year. They should have different levels of maturity.” (AC12_SAP_U4)*
Higher cost of study and its’ value (continued)	Wasting time and lost opportunity	AC11_PSc_U5, AC12_SAP_U4, EXP4_PSc	*“IP-PD student study for 6 year in laboratory and train for R&D but they work in community pharmacy due to the higher salary. They did not use their knowledge and their specialty. It wasted their time and opportunity to work because they have to study and training for one more year instead of working,” (AC11_PSc_U5)*
			*“Improving pharmacist’s competency is a great thing but we have many ways to improve it, such as, via studying or working in real life situation. I did not see the benefit of the extended for one year or the worth of this change. Why it should not be Master degree instead of bachelor degree. Parents have to support cost for another year and social lost pharmacist for another year.” (AC12_SAP_U4)*
			*“They loss opportunity to work. If they graduate in five year, they will work faster for 1 year.” (EXP4_PSc)*
	A 5-year programme still need in some areas	EXP4_PSc, EXP10_HOS, PM10, PHY7	*“A 5-year programme in Pharmaceutical had enough and appropriate basic knowledge to work as pharmacy professional.”(EXP4_PSc)*
			*“I think they are suitable to work in hospital setting but detailer might not need 6 years or production line in hospital might need a 5 year programme.” (EXP10_HOS)*
			*“I want it to be a voluntary PharmD, rather than all six year PharmD. Who want to learn six year, they can learn. Five year curriculum is also need.” (PM10)*
			*“I think the 6 year programme probably too much due to their services might not need to study for 6 years. Most of them might not work with patient in the hospital. Some pharmacists work at community pharmacy that might need the different competencies.” (PHY7)*
	Thai context different from the US context	AC16_SAP_U2, PM2, PM9, PM10	*“I do not see the benefit of the extended one year or the worth of this change. Why should it not be a master’s degree instead of a bachelor degree? Parents have to support the cost for another year and society has lost a pharmacist for another year.” (AC16_SAP_U2)*
			*“We are not prosperous but we are not very poor either. We should undertake appropriate education. The issue is that we would like to follow the western model. We can find our own way. I do not think six years is the best study programme in the world.” (PM10)*
Mismatch between the pharmacy graduates’ competencies and the requirements of the job market	Mismatch competency and market’ needs within pharmaceutical care tract (e.g. hospital and community pharmacy settings)	EXP1_COM, EXP2_COM	*“In the past, the basic skill of Thai pharmacist is able to work as a community pharmacist. Now, the responsibility of community pharmacy had totally changed. Within PC-PD programme, it should be separated into hospital pharmacist and community pharmacist because they have different required competencies.” (EXP 1_COM)*
			*“Some faculties taught their PC-PD graduates that they should work in hospitals only. Students told me that they were not interested in community pharmacy but came here because it is only a compulsory rotation.” (EXP2_COM)*
	Mismatch competency and market’ needs within industrial pharmacy tract	AC10_PSc_U5, AC11_PSc_U5, EXP1_COM, EXP5_COM, PM9	*“The private sector hired graduates from other other areas such as scientists who have a PhD degree. If you compare pharmacists who have a bachelor’s degree and scientists who have a PhD. pharmaceutical companies need PhDs more than pharmacists because they have more experience in R&D” (AC10_PSc_U5)*
			*“Most of the pharm sciences students worked in community pharmacies because they got higher salaries than industrial plants but they were not well trained.” (AC11_PSc_U5)*
			*“PSc-PD graduates should not do practice in community pharmacies unless they have been passed the training and gained skills and competency to qualify to work as community pharmacist.” (EXP1_COM)*
			*Although IP-PD graduates can work in other services such as community pharmacy but they can work in certain levels. It will be difficult when they work in advanced services. Community pharmacy career is improved the services. It will provide the services to chronic and complex diseases patients. IP-PD graduates will struggle." (PM9)*
	Number of IP-PD was higher than job market needed	AC1_PSc_U1, PC10_PSc_U5, AC13_PC_U4, AC21_D_U5, AC22_D_U1, PM7, PM9	*“The number of IP-PD students was higher than job market need. I knew the job market. I feel empathy for students.” (AC1_PSc_U1)*
			*“There is limited job position in pharmaceutical industrial. They need only 200 pharmacists per year. However, it still has pharmacy shortage. I do not know the reason why graduates do not want to work in industrial." (PM7)*
			*“The number of pharm sc graduates should be reduced.“ (PM9)*
Mismatch between the pharmacy graduates’ competencies and the requirements of the job market.	Capacity of production depends on supply side	AC1_PSc_U1, AC15_PSc_U2, AC21_D_U5, PH1_HOS, PM4, PM_6, PM9	*“This is problem in a level of policy. We might not base on principles of market demand.” (AC1_PSc_U1)*
			*“We have a readiness and preparedness in industrial pharmacy more than clinical pharmacy so we have the number of IP-PD more than PC-PD.”(AC15_PSc_U2)*
			*“Basically, our basic structure is mainly in pharmaceutical sciences area. Number of pharmaceutical sciences academic staff higher than Pharm care staff. So, we have high number of student in IP-PD tract because we have to consider about education management.” (AC21_D_U5)*
	IP-PD graduates have a competitor in the job market	AC11_PSc_U5, EXP7_PB, PM7	*“Pharmaceutical company need PhD more than pharmacist because they had more experience in R&D.” (AC11_PSc_U5) (Note: PharmD degree in Thailand equivalent to Bachelor degree)*
			*“The opportunity of the 6-year IP-PD students would be less because manufacturers might hire a 4-year scientist instead. It might save cost for manufacturers. Pharmacist will have competitor in the job market.” (EXP7_PB)*
	Need more communication	AC1_PSc_U1, AC3_PC_U4, AC9_SAP_U1, AC12_SAP_U4, AC13_PC_U1, AC21_D_U5, AC22_D_U1, EXP4_PSc, EXP5_PSc, PM1, PM4, PM5, PM10, ST1	*“I feel uncomfortable about this change. It needed more consideration before creating this new curriculum. It needed communication to answer questions such as the necessity for change, the direction of change, and the main barrier that should be considered.” (AC13_PC_U1)*
			ᅟ
			*“There was confused about Pharmaceutical sciences. What is their scope and competency? Someone said all work which is non-pharm care is pharm sciences. We have to discuss about their competencies, regarding to the need of users.”* (EXP5_PSc)
			*“It's a top down policy of the powerful people at that time. It should have more serious consideration of views or listening to each other.”* (PM10)
Lack of resources	Too quick and not ready	AC1_PSc_U1, AC2_PC_U4, AC3_PC_U4, AC10_PSc_U5, AC12_SAP_U4, AC15_PSc_U2, AC21_D_U5, EXP5_PSc, PM2	*“Everything was move too fast. Even policy is suite for the future but we have no personnel to serve the policy. We should prepare PharmD preceptors then change to an entry level PharmD.”(AC3_PC_U4)*
			*“There was many problems such as preceptors were not ready, academic staff also were not ready.” (EXP5_PSc)*
			*Contrast view*
			*“We have been informed about this change for many years before the PCT announced.”(AC15_PSc_U2)*
			*“It seems like question of “Which came first the chicken or the egg?” If preceptors are not ready, we should train them. If preceptor is not enough, we have to increase the number of preceptor.” (PM2)*
	Lack of preceptors and training sites	AC2_PC_U4, AC5_SAP_U1, AC9_SAP_U1, AC10_PSc_U5, AC13_PC_U1, AC18_PC_U3, AC19_PC_U3, AC20_PC_U3, AC22_D_U1, EXP3_HOS, EXP4_IP, EXP5_IP, EXP7_PB, EXP8_PB, EXP9_HOS, EXP12_COM, PH3_HOS, PH9_HOS, PH6_HOS, PH7_HOS, PH10_HOS, PH11_HOS, PH12_HOS, PH15_COM, PH16_COM, PH20_IP, PH21_IP, PH24_HOS, PH25_HOS, PM3, PM4, PM9, PM10	*“We have to have our own staff to teach our students at practice sites because pharmacists have high workload. We have MOU with hospitals and have a great support from both faculty and hospital administrators.” (AC5_SAP_U1)*
			*“It increases the load of preceptors, of which they are still the same number. Every faculty is competing.” (AC22_D_U1)*
			*“Increasing to the 6-year programme and release students out into the real world to find experience for themselves is not the right way. Lecturers or master’s degree students should be trained and transfer their experience to students. At least 20 students will be trained at each site, but the site employs only one pharmacist.” (PM4)*
	Plan to develop preceptors and teacher practitioners	AC3_PC_U4, AC5_SAP_U1, AC18_PC_U3, AC19_PC_U3, AC20_PC_U3, AC21_D_U5, AC22_D_U1, AC23_D_U2, AC24_D_U3, AC25_PC_U3, EXP3_HOS, EXP4_IP, EXP5_IP, EXP7_PB, EXP8_PB, EXP13_HOS, PH9_HOS, PH19_RD, PM9	*“Newly PhD or young staff are work hard for faculty but they did not have time to do their own career ladder. Leaders have to protect them. We should have a good mentor for them.” (AC3_PC_U4)*
			*“The most important factor in the new programme is preceptors and teacher practitioners. We have teacher practitioners (TP) to work in hospital as teacher for students and also practice at training site.” (AC5_SAP_U1)*
	Good attitude of preceptors	AC2_PC_U4, AC8_PC_U4, PH2_HOS, PH6_HOS, PH10_HOS, PH11_HOS, PH24_HOS	*“Training pharmacy students is my responsibility. I am a pharmacist and I want to make the pharmacy profession stronger. I am proud to be a preceptor.” (PH24_HOS)*
	Barriers to be a preceptor (e.g. workload, time)	AC10_PSc_U5, EXP6_PB, EXP13_HOS, PH25_HOS, PH16_COM, PH17_RD, ST8, PH021_IP, PH23_PB, PH30_COM	*“Being preceptor took a pharmacists’ time.” (EXP13_HOS)*
			*“You want an effective preceptor but you never train us. Is it too demanding? We already have a high workload.” (PH25_HOS)*
			*“Some of them might not ready to train us because of they did not have enough time due to their high workload.” (ST8)*
Lack of resources (continued)	Lack of academic staff in pharmaceutical care area	AC2_PC_U4, AC4_PC_U3, AC9_SAP_U1, AC10_PSc_U5, AC21_D_U5, AC25_PC_U3, PM9	*“We need real expertise staff in pharm care that they should have residency degree or board certify but there were a very low number of this group of academic staff.” (AC21_D_U5)*
			*“Pharmacy graduates in pharm care preferred to work in the hospital more than to be a lecturer because of high salary and less stress. We are lack of lecturers and teacher practitioners.” (PM9)*
	Lack of practical experience of the faculty members in both pharmaceutical care and pharmaceutical sciences	AC10_PSc_U5, EXP1_COM, EXP2_COM, EXP5_IP, EXP13_HOS, EXP7_PB	*“Most of academic staff works on their research but rarely work in real practice settings.” (EXP7_PB)*
Separate licences	Specialty competency should have separate license	AC12_SAP_U4, EXP1_COM, EXP2_COM, EXP5_PSc, EXP7_PB, PM1, PM5, PM6	*“I encourage the separate license because each specialty required different competency. For example, within pharmaceutical care, there should separate into hospital pharmacist and community pharmacist. IP-PD should not practice in community pharmacies unless they pass the training and qualify to work as community pharmacist.”(EXP1_COM)*
			*“Pharmacy professional in each track will be developed. In far future, there is might be a separation of licensed." (PM1)*
			*“The big issue was relating to license, but at that time, the council is not ready to give two licenses. As a result, every university have to change to six-year curriculum with one license.” (PM5)*
			*“Separate licences seems to narrow down pharmacists’ opportunities to work in various settings. However, we need to be specialised, which requires different competencies. So, there should be separate licenses.” (PM 6)*
			*Contrast view*
			*“I did not agree about separate license. Why we have to close our opportunity? (EXP5_PSc)*
			*ᅟ*
	Different programme and two examination	AC9_SAP_U1, AC10_PSc_U5, AC11_PSc_U5, AC14_SAP_U5, PM6, PM9	*“If they study the difference programmes from first year, they should have difference license.” (AC9_SAP_U1)*
			*“We will have two pharmacy licensure examinations. The first examination is for the core competency. The second examination is for the specialty. Why we have just only one license?” (PM6)*
	Competencies of the entry level pharmacy graduates	EXP1_COM, EXP2_COM, EXP6_PB, EXP13_HOS, PM1, PM3, PM10	*“The confusion in our pharmacy profession that we never talk is about the in- depth of core competency in the entry level PharmD programme.” (EXP6_PB)*

^a^Abbreviation

*AC_PSc* Academic member in pharmaceutical sciences area, *AC_PC* Academic member in pharmaceutical care area, *AC_SAP* Academic member in social and administrative pharmacy), *AC_D* Dean, *EXP_HOS* Expert in hospital pharmacy, *EXP_COM* community pharmacy, *EXP_PSc* Expert in industrial pharmacy, *EXP_PB* Expert in public health and consumer protection, *EXP_MP* Expert in marketing pharmacy, *N* Nurse, *PH_HOS* Hospital pharmacist, *PH_COM* community pharmacist, *PH_IP* Industrial pharmacist, *PH_PB* Pharmacist in public health and consumer protection, *PH_MP* Pharmacist in marketing pharmacy, *PH_RD* Pharmacist in Research and development, *PHY* Physician, *PM* Policy maker, *PTECH* Pharmacy technician, *PT_IPD* Patient in In-Patient Department, *PT_OPD* Patient in Out-Patient Department, *PUB* Public/general population, *ST* Student, *PR* Parent, *U1* University1, *U2* University2, *U3* University3, *U4* University4, *U5* University5

### Higher cost of study and value

Some academic staff thought that the policy makers should consider the increased cost of study due to the extra year. The students also discussed whether the increased cost of study was worth it.

*“The negative effect is the cost of education to parents and the government budget of at least six hundred million baht (£12,253,000 or $18,421,000) a year, without being able to see the benefit in the short or long term. Improving pharmacists’ competencies is a great thing but we have many ways to improve them, such as via studying or working in real life situations.” (Academic member 12)*

(Note: exchange rates on 08/03/2015; 1 GBP = 48.967 THB and 1 USD =32.570 THB)

*“The Pharmacy Council should prepare salary or compensation for our degree. I heard that they increased our salary by only 1,000 baht (£20 or $31) a month compared to the 5-year programme. It is not worth it to study another one year more, when we have to spend approximately 100,000 baht (£2042 or $3070) for this extended year. There should be other ways to encourage us to work hard and deserve our salary.” (PharmD student 1)*

Some interviewees were concerned that this change might not deliver an effective pharmacy curriculum that is suitable for Thai context. They suggested that the new curriculum might cause problems in terms of students’ time and money and might limit the ability to produce pharmacists who could quickly begin to work for Thai society.

*“We are not prosperous but we are not very poor either. We should undertake appropriate education. The issue is that we would like to follow the western model. We can find our own way. I do not think six years is the best study programme in the world.” (Policy maker 10)*

*“I do not see the benefit of the extended one year or the value of this change. Why should it not be a master’s degree instead of a bachelor degree? Parents have to support the cost for another year and society has lost a pharmacist for another year.” (Academic member 16)*

Parents of pharmacy students who studied at private universities said that the cost of their children’s education was at least 100,000 baht/year and approximately 500,000 baht *(£10,200 or $15,300)* in the 6^th^ year; the total cost was approximately 1,600,000 baht *(£32,600 or $49,100)*. One father thought that the cost might cause problems for some families who had low to medium incomes, but for him, it was acceptable.

*“I think the cost is quite heavy. In cases where we do not have money, this might be a problem.” (Parent 1)*

Another interviewee, from a family with a low income, thought the cost of education at public university of approximately 40,000 baht/year (£800 or $1,200), and that the total cost, which was approximately 300,000 baht (£6,100 or $9,200), was acceptable. Her son was granted a scholarship from his faculty and he was very careful with his money and also had a part time job for his living expenses. However, in terms of the cost of education, even at a public university, it is still a large amount of money for students’ families.

*“The cost is not much harder because he (her son) helps himself. He got a scholarship from the faculty and works part-time job with his teacher. When he comes back home, he rarely asks for money. He needs only approximately 5,000 baht (£100 or $150) per month. He is a very good saver. He rarely buys new clothes.” (Parent 3)*

### Mismatch between the pharmacy graduates’ competencies and the requirements of the job market

The traditional PharmD programme focused on patient care but the PharmD curriculum in Thailand is divided into two main streams: a pharmaceutical care-PharmD (PC-PD) programme and a pharmaceutical Sciences or industrial pharmacy-PharmD (IP-PD) programme. However, both give students the same license to work across all sectors. There is a general consensus among the interviewees that the aim of the development of a 6-year programme in Thailand was to improve pharmacy competencies from generalists to specialists, focusing on pharmaceutical care and preparing pharmacy graduates to practice upon graduation in real workplace settings. Such preparations would serve to meet the required needs of the stakeholders. The greatest benefits of producing competent clinical pharmacists should be to patients, health consumers and Thai society.

However, community pharmacy employers presented contrasting views. Some argued that the pharmaceutical care aspect of the PC-PD is very hospital-centred and the skills acquired are more suited for tertiary and secondary care settings than primary hospital and community pharmacy settings.

*“Within the PC-PD programme, it should be separated into hospital pharmacist and community pharmacist because they have different required competencies.” (Pharmacy expert 1, community pharmacy)*

*“Some faculties taught their PC-PD graduates that they should work in hospitals only. Students told me that they were not interested in community pharmacy but came here because it is only a compulsory rotation.” (Pharmacy expert 2, community pharmacy)*

Pharmacists and pharmacy experts from the consumer protection area also shared the opinion that the curriculum should prepare pharmacy graduates for primary care services due to the higher number of primary care hospital settings (community hospitals and sub-district health-promoting hospitals).

On the other hand, the majority of the interviewees were less positive about the IP-PD graduates meeting the needs of the market; the number of graduates currently exceeds the needs of industry, and graduates from this track often have to pursue career paths in community pharmacy, a sector for which they are not prepared.

*“Most of the pharmaceutical sciences students worked in community pharmacies because they got higher salaries than industrial plants but they were not well trained.” (Academic member 11)*

In addition, this view is also shared by participants from the industrial sector who believe that IP-PD graduates lack the research skills required for the research and development industry and often prefer to employ PhD holders of non-pharmacy science backgrounds.

*“The private sector hired graduates from other areas such as scientists who have a PhD degree. If you compare pharmacists who have a bachelor's degree and scientists who have a PhD, pharmaceutical companies need PhDs more than pharmacists because they have more experience in R&D.” (Academic member 10)*

In contrast, some policy makers perceived that the PharmD graduates will be more skilled in research and development, which might meet the needs of employers. There are plenty of opportunities to create the curriculum and make a difference for graduates, but this depends on the cooperation of the leader, dean and academic staff in the pharmaceutical sciences area to prepare this new curriculum.

*“Job markets are still available but we have to open new markets, such as areas in vaccines, blood substitutes, nanotechnology, in herbal medicine. Faculty should move to design our graduates.” (Policy maker 5)*

To fulfil the needs of the IP-PD graduates’ competency for the area of research and development, an integrated PharmD-PhD programme was developed at one of the universities in Thailand, aiming to prepare highly competent graduates to study in PhD programmes. This is an interesting programme designed to suit the needs of the pharmacy or pharmaceutical industry.

### Lack of preceptor and training sites

The crucial issue for the transition to an all-PharmD programme is providing a sufficient number of qualified PharmD preceptors. The new PharmD curriculum has a four-fold increase in the number of hours for practice training compared with the BPharm programme. However, the number of qualified preceptors remains the same. The PECT tried to establish a preceptor development programme to prepare for this change but the majority of the stakeholders perceived that this was still not enough.

*“It increases the load of preceptors, of which there are still the same number. Every faculty is competing.” (Academic member 22)*

Stakeholders felt there were benefits for institutions if they are offered as training sites (e.g., contributing to the pharmacy profession, updating preceptors’ knowledge and skills, having highly competent academic members who are able to empower preceptors and enhance training sites and opportunities to recruit well-performing pharmacy students).

*“Training pharmacy students is my responsibility. I am a pharmacist and I want to make the pharmacy profession stronger. I am proud to be a preceptor.” (Pharmacist 24)*

However, the majority of the interviewees had common concerns regarding the insufficient quantity and quality of preceptors. Stakeholders’ perceived barriers towards formal preceptor preparation, such as the workload (e.g., high routine workload of the preceptors, lack of time/money/management staff/space), inadequate role models, the need for more recognition and support from administrators regarding preceptors’ roles, training sites requiring standardisation and quality assurance, career progression as preceptors and a reward system for their clerkship workload, the need to put in place a preceptor development programme and the establishment of an active memorandum of understanding (MoU)/long term commitment between training sites and universities.

*“You want an effective preceptor but you never train us. Is it too demanding? We already have a high workload.” (Pharmacist 25)*

One employer in industrial pharmacy thought that the training patterns in the pharmaceutical sciences area should be reconsidered and needed changing. Academics who have more training in research should teach students about pharmaceutical sciences in the universities. This strategy might be better than sending students to be trained in industry, which has a limited number of preceptors, insufficient space and too few training sites. Of more concern, some trainers who were non-pharmacists in industrial pharmacy settings were diploma graduates who wondered about their qualification to teach pharmacy students.

*“Increasing to six years and releasing students out into the real world to find experience for themselves is not the right way. Lecturers or master's degree students should be trained, and their experience will transfer to students. At least 20 students will be trained at each site, but the site employs only one pharmacist.” (Policy maker 4)*

### Lack of practical experience of the faculty members

Deans and policy makers had planned to increase the number of instructors in the pharmaceutical care area due the lack of pharmacy practice staff within academic institutions. Unfortunately, they found it was difficult to recruit pharmacy graduates to work as instructors in pharmaceutical care within academic institutions because they preferred to work in other pharmacy practice areas such as hospital or community pharmacy settings due to higher salaries and less stressful environments.

*“Pharmacy graduates in pharm care preferred to work in the hospital more than to be a lecturer because of higher salary and less stress.” (Policy maker 9)*

Highly performing academic staff in the pharmaceutical care area who have graduated from a pharmacotherapy residency programme are scarce and are in great demand. They also act as role models for PharmD students. Senior academics and experts said that they work very hard and that faculty have to take care of them and not let them become burnt out by the high workload.

### Separate licences

Previously there has only been one type of license for Thai pharmacists across all pharmacy practice settings. Pharmacy graduates from both the BPharm and PharmD programmes have been required to take the same national licensure examination. However, the PharmD students who start their pharmacy education in and after 2015 will have to take two pharmacy licensure examinations: the first examination is for pharmacy core competency at the end of their fourth year and the second examination is for their specialised competency at the end of their sixth year. The examination had to be separated into two different examinations because those two tracks were very different in terms of their specialties (e.g., knowledge content, clerkship experiences and specialised skills) but they still have the same license. Separate licenses were mentioned in earlier stages of decision making.

Some faculty members were concerned about the vision of the Thai pharmacy profession. If it aimed to move practitioners from being generalists to specialist pharmacists, different types of pharmacy licenses should be offered. Some policy makers advised to offer separate pharmacy licences in the future, and pharmacists should not work across the professional pharmacy tracks; for instance, pharmacy graduates in pharmaceutical industrial should not work in community pharmacy because they may not have sufficient competency. Pharmacy professionals in specific areas should work only in their area because this represents a commitment to the development of the pharmacy profession in those specific areas. They might cross over to other tracks, but there should be a system to assess their competencies (e.g., taking a training course or continuation of their pharmacy education) to ensure the delivery of good pharmacy practice and to meet the required standard in each practice area.

*“Separate licences seems to narrow down pharmacists’ opportunities to work in various settings. However, we need to be specialised, which requires different competencies. So, there should be separate licenses.” (Policy maker 6)*

On the other hand, some academics said that there should be only one license. They thought that the advantages were as follows: an opportunity to work in various areas of pharmacy practice, as in the past, and one license would, or at least could, unite the pharmacy profession. The situation was frequently compared to the doctor’s license: a doctor has only one license but different doctors might have different specialties.

## Discussion

In the past three decades, the roles of pharmacists globally have changed dramatically. Pharmacists responsibility are not merely in compounding or dispensing medicines but in providing a professional role in patient care [7]. The main limitation to developing advanced clinical roles is a lack of clinical skills. The genesis of the 6-year PharmD programme in Thailand began from the needs of pharmacists who would like to develop themselves to provide a better standard of patient care. It was similar to the adoption of the PharmD in the US, Canada and South Korea that was led by the needs of clinical pharmacists who had higher competency in patient care [10, 89, 90]. The PharmD programme is the model for the pharmaceutical care programmes employed by many other countries [11]. It has been mentioned in many countries who have adopted, or plan to [8] adopt, this programme to produce pharmacy graduates who have high levels of knowledge and skill in pharmaceutical care and who work well together with other health care providers [7, 91, 92].

The strong cooperative network of PECT and the US-Thai consortium for the development of pharmacy education in Thailand appeared to be the most important influence on the development of the PharmD programme in Thailand [18, 20–22]. Another important influence is the regulatory body that has the authority to make the policy a reality. The process of the ‘all-PharmD’ programme has also been mandated by the authorities in many countries (e.g., Canada, Japan, South Korea) [7, 10, 93].

Interviewees in the pharmaceutical care area appeared to welcome the 6-year PharmD programme due to this new programme being a step-up process for the pharmacy profession that has the same curriculum duration as the MD or DDS degree, filling the gap left by the 5-year BPharm programme, which involved less practice and might produce a graduate who had insufficient competencies to practice on the job market today. This benefit was also mentioned in the adoption of a 6-year pharmacy programme in the US and South Korea [8, 94]. Another important benefit is that PharmD graduates are ready to work, with little support, following graduation and thus are becoming increasingly common and valued in the Thai health system [21, 95, 96].

Thailand is among the world’s middle income countries. An average monthly income per household in the whole Kingdom of Thailand in 2013 was 25,194 baht (£477 or $746) [97]. The expenditure for one full time student at faculty of pharmacy of a public university was 140,000 baht per year (£2,700 or $4,100) [98]. The proposed increased average cost to cover the additional one year in the PharmD programme was 600,000,000 baht (£11,590,000 or $17,780,000) [48]. Some parents in this study and others reported concerns about the cost of pharmacy education, which can be a major financial investment. However, they were prepared to support their students or seek other financial support such as grants, scholarships or government-sponsored student loan schemes [99–101]. Some academic staff were concerned that the increase of an extra year of education would limit the ability of students to start earning an income and might be a burden for students, their families and the government. This study noted the same considerations as another study [100, 101], in that the curriculum should be designed to deliver an effective education that is able to produce competent pharmacy graduates while also saving money and time. Some students were concerned about the fact that tuition have increased fees but salaries and available positions have not increased, which was a similar concern in the US [100]. Cain et al. suggested that it is necessary to ensure that excellent students are not deterred from pharmacy education by concerns about insurmountable costs or debt after graduation [100].

There were also concerns about the insufficient quantity and quality of trained preceptors, multidisciplinary learning and practice in training sites, quality assurance, support and recognition from administration, and a preceptor development programme [102, 103].

The Thai and Pakistani PharmD curricula are both different from the US PharmD [37, 104]. They were adapted to meet the country’s needs by including clinical tracks and industrial pharmacy tracks. In 2012, the percentages of Thai pharmacists in each sector were as follows: hospital (40 %), pharmacy marketing (22 %), community pharmacy (17 %), pharmaceutical industry (10 %), consumer protection (6 %) and education (5 %) [5, 105]. Hence, a single pattern pharmacy programme might not be able to produce all of the competencies needed in all of the pharmacy practice areas [3, 5]. This study found a mismatch between pharmacy graduates’ competencies and the requirements of the job market. Competencies required for an industrial pharmacist are completely different from a pharmacist who provides pharmaceutical care in tertiary hospitals or from the community pharmacist who provides home health care and health promotion [23, 37]. It is interesting that most pharmaceutical sciences track graduates appeared to work in the community pharmacy setting. Academics should seriously revise the content and practical experiences in the curriculum to meet the needs of graduates and Thai society [1, 3, 106]. Faculties should coordinate with the various Thai pharmacy associations, such as the Association of Hospital Pharmacy (Thailand), Community Pharmacy Association (Thailand), Thai Industrial Pharmacist Association, and Marketing Pharmacy Association of Thailand to update the competencies required by pharmacy graduates. Faculties must adapt more quickly (their vision, academic workforce planning, providing facilities, preceptor co-development programme, legal considerations affecting pharmacists’ roles and responsibilities) to support the needs of society and rapidly changing health care systems [1, 7, 39, 93, 104].

This study might be of interest to faculties and policy makers to develop pharmacy curricula and national pharmacy competency standards to produce future pharmacy practitioners and pharmaceutical scientists who are ready to work to deliver high-quality services to patients and the public and also consider national pharmacy workforce planning to propose numbers of prospective pharmacy students in different specialties.

The limitation of this study lies in the volunteer participants. Some informants who were involved in the transition process did not participate in this study. It is unclear whether there are differences in the experience and perceptions of the two groups of informants: those who decided to participate and those who did not participate in this study. The findings might be influenced by some participants’ enthusiasm about this transition [107]. However, this study attempted to include a maximum variation of participants [65], including those who agree and do not agree with this curriculum change. Another limitation is that the transition to an all-PharmD programme is still at an early stage. The first cohort of “all-PharmD students” graduated in March 2015, so the benefit or merit of this transition in terms of both education and services might not be clearly visible at the time of this study (2013). More prolonged and in-depth study is needed to determine the impact of the transition on students' competencies, professional performance in pharmacy services and the satisfaction of employers and society [107].

## Conclusions

This is the first study of its kind to highlight the issues surrounding the transition to the 6-year PharmD programme in Thailand, which was initiated due to the need for higher levels of competency for the nation’s pharmacists. The transition was influenced by many factors (e.g., the global trend towards providing patient care services, cooperation of four pharmacy faculties and the Ministry of Public Health to develop a foundation for clinical pharmacy activities in hospital settings, the US-Thai consortium for the development of pharmacy education, which founded by the PECT, and the establishment of the PCT). Many participants perceived benefits from the new pharmacy curriculum; for example, the PharmD graduates will acquire an advanced pharmacy professional degree, improve pharmacy competencies from generalists to specialists, will be ready to work as pharmacists after graduation, will understand health care teams and will provide a high quality of patient care. However, some participants were concerned regarding the curriculum change, such as regarding the higher costs of a longer period of time for study, the mismatch between the pharmacy graduates’ competencies and the job market’s needs, the need to consider designing the curriculum to suit pharmacy services in other areas in addition to tertiary care settings (e.g., primary care, community pharmacy, consumer protection), and that the number of graduates in IP-PD might exceed the needs of industry, leading them to pursue career paths in community pharmacy for which they were not well prepared. The most crucial concerns are about the insufficient preceptors and training sites, the lack of practical experience of the faculty members and issues related to separate licenses due to the differences in graduates’ specialties. Although most of the respondents accepted the need to go forward to the 6-year PharmD programme, the design of an effective curricular, providing a sufficient number of qualified PharmD preceptors, determining certain competencies of pharmacists in different practices and monitoring the quality of pharmacy education still need to be addressed in this transition stage of pharmacy education in Thailand.

## Abbreviations

DDS: Doctor of Dental Surgery
DVM: Doctor of Veterinary Medicine
FIP*Ed*: International Pharmaceutical Federation Education Development Team
IP-PD: Industrial Pharmacy-PharmD programme
MD: Doctor of Medicine
MoPH: Ministry of Public Health
PC-PD: Pharmaceutical care-PharmD programme
PharmD: Doctor of Pharmacy
PECT: The Pharmacy Education Consortium of Thailand
PCT: The Pharmacy Council of Thailand
SAP: Social and Administrative Pharmacy
WHO: The World Health Organization
UNESCO: The United Nations Educational, Scientific and Cultural Organisation
US: The United States of America
